# Survival of hereditary non-polyposis colorectal cancer patients compared with sporadic colorectal cancer patients

**DOI:** 10.1186/1756-9966-27-39

**Published:** 2008-09-19

**Authors:** Vittoria Stigliano, Daniela Assisi, Maurizio Cosimelli, Raffaele Palmirotta, Diana Giannarelli, Marcella Mottolese, Lupe Sanchez Mete, Raffaello Mancini, Vincenzo Casale

**Affiliations:** 1 Gastroenterology and Digestive Endoscopic Unit, Regina Elena Cancer Institute Via Elio Chianesi 53, 00144 Rome, Italy; 2 Department of Surgery, Regina Elena Cancer Institute, Via Elio Chianesi 53, 00144, Rome, Italy; 3 Department of Laboratory Medicine and Advanced Biotechnologies IRCCS San Raffaele Pisana, Via della Pisana 235, 00163 Rome, Italy; 4 Biostatistic Unit, Regina Elena Cancer Institute Via Elio Chianesi 53, 00144 Rome, Italy; 5 Department of Pathology Regina Elena Cancer Institute, Via Elio Chianesi 53, 00144 Rome, Italy

## Abstract

**Background:**

Patients with hereditary non-poliposys colorectal cancer (HNPCC) have better prognosis than sporadic colorectal cancer (CRC). Aim of our retrospective study was to compare the overall survival between sporadic CRC and HNPCC patients.

**Methods:**

We analyzed a cohort of 40 (25 males and 15 females) HNPCC cases with a hospital consecutive series of 573 (312 males and 261 females) sporadic CRC observed during the period 1970–1993. In 15 HNPCC patients we performed mutational analysis for microsatellite instability. Survival rates were calculated by Kaplan-Meier method and compared with log rank test.

**Results:**

The median age at diagnosis of the primary CRC was 46.8 years in the HNPCC series versus 61 years in sporadic CRC group. In HNPCC group 85% had a right cancer location, vs. 57% in the sporadic cancer group. In the sporadic cancer group 61.6% were early-stages cancer (Dukes' A and B) vs. 70% in the HNPCC group (p = ns). The crude 5-years cumulative survival after the primary CRC was 94.2% in HNPCC patients vs. 75.3% in sporadic cancer patients (p < 0.0001).

**Conclusion:**

Our results show that overall survival of colorectal cancer in patients with HNPCC is better than sporadic CRC patients. The different outcome probably relates to the specific tumorigenesis involving DNA mismatch repair dysfunction.

## Background

Colorectal cancer is one of the most common neoplasm in humans [1,2]. It's known that a definite fraction, ranging between 1 and 5% of all cases of colorectal tumors, is transmitted from one generation to another in accordance with an autosomal dominant model; this is the case of Hereditary Non-Polyposis Colorectal Cancer (HNPCC) and of Familial Adenomatous Polyposis (FAP) [2].

HNPCC is an autosomal dominant disease characterized by early appearance of cancer usually of the right colon, frequent occurrence of multiple lesions (both synchronous and metachronous) and a striking association with tumours of other organs, in particular endometrium, urinary tract, ovary, stomach and small bowel. The Amsterdam criteria, currently used for the diagnosis of HNPCC, were introduced in 1989 to provide a uniform evaluation of familial and personal history. They were revised in 1999 and included various extra-colonic tumours: 1) at least three or more relatives with histological verified tumour in the spectrum of HNPCC, one of whom is a first-degree relative of the other two; 2) at least two generations should be affected; 3) one or more tumours diagnosed when under the age of 50 years; 4) FAP should be excluded [3,4].

Colorectal tumours with microsatellite instability (MSI phenotype) have mutations in mismatch repair (MMR) genes and in particular in the MSH2 and MLH1 genes, respectively found on chromosome arms 2p and 3p [5-9]. These mutations lead to inactivation of the genes and thus to a defect in replication/repair of DNA and an accumulation in the cancer cell genome of ubiquitous somatic clonal mutations [10]. Constitutional mutations of MMR genes are found in 50–70% of hereditary non-polyposis colorectal cancer (HNPCC) and in the Muir-Torre syndrome; 90% of them MLH1 and MSH2 positive [11,12]. A certain proportion (around 12–15%) of sporadic colon cancers also display MSI phenotype [13-17]. Otherwise, MSI seems to be important in the development of various human cancer such as sporadic endometrial cancer [18] and oral squamous cell carcinoma [19].

Colorectal cancer (CRC) in HNPCC more often have a better prognosis than in sporadic colorectal carcinoma [3,4,20-22], but it has been unclear whether this could be due to difference in stage at diagnosis or to a more favourable prognosis of cancer in HNPCC and FAP.

The aim of our retrospective study was to compare stage and overall survival between patients with hereditary and sporadic colorectal cancer patients.

## Methods

We analyzed 40 HNPCC patients (25 males and 15 females) with histological verified colorectal carcinoma and a consecutive series of 573 (312 males and 261 females) sporadic CRC patients with no familial predisposition, observed and treated at the Regina Elena Cancer Institute of Rome during the period 1970–1993 [23]. HNPCC patients were selected by both personal and familial history (performing complete pedigree including first and second-degree relatives) according to Amsterdam I criteria. In 15 patients of the HNPCC group, we performed mutational analysis for microsatellite instability, investigating mutations in mismatch repair genes as hMLH1 and hMSH2 correlated with disease. We analyzed seven microsatellite loci: D2S123, D3S1611, and BAT-26, D9S145, D1S158, SCZD1, and D11S905 [5,14,24,25].

All cases were pathologically staged taking into account a total number of lymphonodes exceeding 7 (range 7 to 31, mean 13.0, mode 11.9, standard deviation 6.2), as recommended by the American Joint Committee on Cancer/International Union Against Cancer [26].

Histological examination was performed according to WHO criteria and carcinomas were classified according to the Dukes' stage. Furthermore, we defined the right colon as the tract from the cecum to the splenic flexure. The left colon included the descending and sigmoid colon. The rectum was defined as the rectosigmoid junction and rectum.

From 1990, the rectal cancer patients clinically staged Dukes' B and C were addressed to high-dose pelvic radiotherapy, all the rectal cancer patients underwent a total mesorectal excision (TME) surgery. Adjuvant i.v. chemotherapy was administered in all the eligible Dukes' C colorectal cancer patients, those staged Dukes' D underwent chemotherapy.

Patients with sporadic CRC were submitted to a yearly colonoscopy for the first 5 years and every 2 years thereafter [23], HNPCC patients with CRC were submitted to a yearly colonoscopy. Considering the increased risk of extracolonic manifestations, hereditary colorectal cancer patients were also submitted to periodical instrumental examinations tailored to the different spectrum of the disease [4,27].

The index date for survival calculation was defined as the date of treatment for the first colorectal cancer. Clinical follow-up procedures provided information on the subject's status.

### Statistical analysis

Survival curves were estimated using the Kaplan-Meier method and stratified according to various clinical and pathological variables. Differences were tested using the log-rank test. Multivariate analysis was performed using the Cox regression analysis.

## Results

The median age at diagnosis of the primary CRC was 46.8 years in the HNPCC group versus 61 years in sporadic CRC group. In the HNPCC group 34 patients (85%) had colon tumours and 6 (15%) had rectal cancer. In sporadic colorectal cancer group 326 (57%) had colon tumors and 247 (43%) had rectal cancer (p = 0.0001) (Table 1).

**Table 1:** Distribution of colorectal cancer by site

**Tumour site**	**HNPCC**	**Sporadic CRC**
Colon	34 (85%)	326 (57%)
Rectal	6 (15%)	247 (43%)

*P = 0.0001

In the sporadic cancer group 353 (61.6%) had an early-stage cancer (94 Dukes' A and 259 Dukes' B) and 220 (38.4%) had an advanced carcinoma (123 Dukes' C and 97 Dukes' D). In the HNPCC group 28 (70%) had an early stage cancer (9 Dukes' A and 19 Dukes' B) and 12 (30%) had an advanced carcinoma (10 Dukes' C and 2 Dukes' D). The difference was not statistically significant (p = 0.29) (Table 2).

**Table 2:** Distribution of colorectal cancer by Dukes' stage

**Dukes' Stage**	**HNPCC**	**Sporadic CRC**
A	9 (22.5%)	94 (16.4%)
B	19 (47.5%)	259 (45.2%)
C	10 (25%)	123 (21.4%)
D	2 (5%)	97 (16.9%)

The presence of synchronous or metachronous tumours was also investigated for this population. In particular, we did not have more than one cancer at the first diagnosis in either the HNPCC group or sporadic CRC group. Metachronous tumours were observed in 4/40 (10%) of HNPCC tumors and in 10/573 (1.7%) of sporadic colorectal cancer (p = 0.001). The median observation time of two groups is respectively 56 months for sporadic CRC and 54 months for HNPCC.

**Figure 1:**
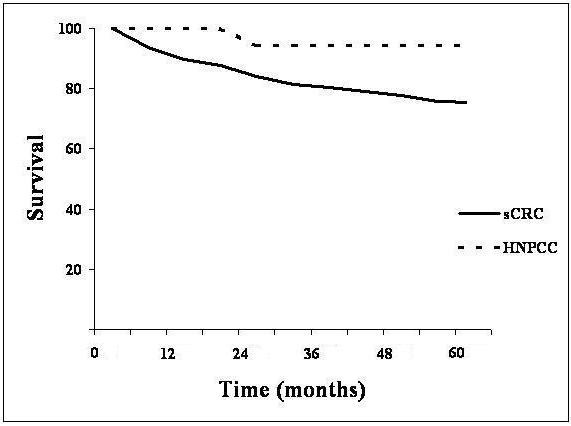
Overall 5-year survival of sporadic colorectal cancer (sCRC) and Hereditary Nonpolyposis Colorectal Cancer (HNPCC).

The 5-years cumulative disease-overall survival after the primary CRC was 94,2% in the HNPCC patients and 75.3% in the sporadic CRC ones (Figure 1) (p < 0.0001). After stratification for Dukes' classification, survival of Dukes' A and B cancers (considered as localized carcinoma) was 84.3% in the sporadic cancer patients and 96.3% in the HNPCC patients. Five year survival for Dukes'C was 63.5% and 92.8% in the sporadic CRC and HNPCC respectively. Survival for Dukes' D was 26.6% in the sporadic CRC but in the HNPCC group we could not estimate survival curves by Kaplan Meier method due to the small number of available patients. After stratification for Dukes' stage survival remained statistically significant, better for HNPCC versus sporadic colorectal cancer (p < 0.0001).

Considering the different localization of tumor, we stratified patients by rectal and colon cancer. The cumulative survival after 5 years of patients with rectal cancer, was 69.7% in the sporadic CRC and 83.3% in the HNPCC patients. In colon cancer we had a cumulative survival of 79.3% and 96.3% in sporadic CRC and HNPCC respectively. The log-rank test stratified by localization was significant (p < 0.0001). When we stratified these two groups of patients by age we did not find any difference in survival.

The multivariate analysis of the HNPCC versus CRC groups, Dukes' stage, tumor site showed that all these parameters independently affected disease-specific survival (p < 0.0001, p < 0.0001 and p = 0.03 respectively). Out of 40 HNPCC patients, 15 were investigated for microsatellite instability (MSI); all resulting positive. Furthermore, the HNPCC patients with MSI (15/40) had a survival of 100%.

## Discussion

Different survival rates of patients with colorectal cancer have been investigated in several studies [20-22,28-31]. The results are sometimes conflicting because of the different pathogenetic mechanism of tumorigenesis between sporadic and familiar types of colorectal syndrome (HNPCC in particular). These differences are probably due to different clinical pathological characteristics of neoplasia and genetic alterations. Two major mechanisms of genomic instability have been identified in sporadic colorectal cancer progression. The first, known as chromosomal instability (CIN), results from a series of genetic changes that involve the activation of oncogenes, such as Ki-ras, and inactivation of tumor-suppressor genes, such as TP53 and APC [32-34]. The second, known as microsatellite instability (MSI), was described in association with hereditary nonpolyposis colorectal cancer [35-37].

The aim of our retrospective cohort study was to compare the survival between patients with sporadic and hereditary colorectal cancer after surgical resection. A Finnish study [30] and a recent Lithuanian study [22] reported an improved prognosis for HNPCC patients compared to sporadic colorectal cancer patients, but an Italian study could not confirm this result [28]. The localization of tumor is an important prognostic factor for survival. In our study right localization (from cecum to splenic flexure) is significantly more represented in the HNPCC group with respect to the sporadic colorectal cancer group (p < 0.0001). This different anatomical distribution between HNPCC and sporadic CRC, confirmed in literature [34,35], is one of the Amsterdam criteria for the diagnosis of HNPCC and determines a better prognosis, being less aggressive.

We have considered survival, stratified by site, in the two groups and we demonstrated that survival for rectal cancer does not differ statistically, so the presence of rectal cancer with a known worse prognosis and high rate of recurrence does not influence survival of sCRC or HNPCC (p = 0.45). The statistically significant difference of survival was for colon cancer (right location), independent of stage at diagnosis, between sporadic and HNPCC cancer (p < 0.0001).

Furthermore, in order to have a better definition of population in the study, we considered several clinical features such as the presence of synchronous or metachronous tumors. None of these features were represented sufficiently enough in either of the groups as to influence survival rates.

The 5 years cumulative survival in HNPCC and in sporadic colorectal cancer was 94.2% versus 75.3% respectively. This difference was statistically significant (p < 0.0001). These results do not confirm the observations previously reported by Bertario et al [31] where 5 years cumulative survival in HNPCC, FAP and sporadic colorectal cancer groups was not statistically different. Otherwise, our results conflicts with those of Barnetson et al [21], in which survival was not significantly different among carriers and non-carriers of MMR mutations in a series of early colorectal cancer patients. However, after stratification for Dukes' stage survival remained statistically significant, better for HNPCC versus sporadic colorectal cancer (p < 0.0001). In our study the stage distribution was not significantly different between the two groups (sCRC versus HNPCC), 61.6% vs. 70% and 38.4% vs. 30% respectively (p = 0.29), demonstrating that it did not influence the overall survival between the two groups.

Myrhoj et al [29] reported an improved prognosis of cancer in patients with HNPCC versus sporadic CRC, but in the HNPCC series included a high proportion of localized tumors and this indicated that the good prognosis was based on a more favourable stage at diagnosis. Several studies described a trend toward prolonged survival and better prognosis in patients with mutations or MSI in HNPCC, revealing the presence of distinct biological features of colon cancer in families with or without mutations [25], though, as above mentioned, it was not observed by other authors [21]. In our study 15 HNPCC patients had positive MSI mutational analysis; overall survival of this series of patients was 100%.

## Conclusion

In conclusion, our findings appear to confirm previous studies [30,31] which detected that an improved survival for colon cancer in HNPCC, compared to sporadic CRC, usually occur. In fact, our series of HNPCC patients reveals a marked overall survival advantage, which persisted also after stratification by Dukes' stage. Therefore, survival of HNPCC patients not always depends on an early diagnosis, but probably also on distinct biologic features. In this context, MSI mutation pattern plays an important prognostic role since colon cancer with MSI has a better prognosis than tumours without MSI. A large series of HNPCC should be examined to confirm this data. Furthermore, we need more information about MSI in all kind of colorectal carcinomas, in order to establish postoperative surveillance thus improving patients' prognosis and allowing clinicians to plan more accurate and targeted therapy.

## Competing interests

The authors declare that they have no competing interests.

## Authors' contributions

The final manuscript has been read and approved by all authors. VS conceived of the study, partecipated in its design and coordination and performed clinical and endoscopic examination. DA draft the manuscript, performed clinical and endoscopic follow-up, collected the data. MC performed surgical interventions. RP carried out the molecular genetic studies. DG participated in study design and performed statistical analysis. MM carried out the microsatellite analysis. LSM helped to draft the manuscript, performed clinical and endoscopic follow-up. RM performed post surgical clinical follow-up and data collection. VC participated in study design and coordination.
